# Calpains as Potential Regulators of Microtubule-Associated Cytokinesis: A Cross-Eukaryotic Unifying Hypothesis

**DOI:** 10.3390/life16071188

**Published:** 2026-07-17

**Authors:** Jennifer C. Fletcher, Mary A. Biggs, Hilde-Gunn Opsahl-Sorteberg

**Affiliations:** 1 United State Department of Agriculture-Agricultural Research Service, Plant Gene Expression Center, Albany, CA 94710, USA; jfletcher@berkeley.edu (J.C.F.); maryamelia@berkeley.edu (M.A.B.); 2 Department of Plant and Microbial Biology, University of California Berkeley, Berkeley, CA 94720, USA; 3 Faculty of Biosciences, Norwegian University of Life Sciences, Post box 5003, 1432 Ås, Norway

**Keywords:** brown algae, calpain, cell division, centrosome, cysteine protease, cytokinesis, microtubule organizing center, red algae

## Abstract

Calpains constitute an ancient, extensive family of calcium-dependent cysteine proteases found in some bacteria and most eukaryotes. They are involved in a wide variety of developmental and cellular processes and are implicated in major human diseases, but whether they share an ancestral or broadly conserved cellular role remains unknown. Beyond their core CysPc catalytic domain, calpains contain diverse domain combinations and can be either cytosolic or membrane bound. Here, we develop the hypothesis that both cytosolic and transmembrane calpains may contribute to cytokinesis through positional anchoring and organization of microtubules (MTs). We propose that during plant cell division, the singular transmembrane calpain DEK1 plays a role in localizing and organizing the array of cortical MTs from the microtubule organizing center (MTOC) and may thereby position the cell division plane, potentially affecting preprophase band placement and subsequent cell plate formation. Similarly, during cell division in animals, their cytosolic calpains may be involved in setting the point of membrane invagination via their association with membrane-bound proteins. We discuss this novel model for calpain activity in the context of data from the animal and plant literature, as well as of our discovery of putative calpain sequences in both brown and red algal genomes. These findings are consistent with the view that calpains were present early in eukaryotic evolution and diversified alongside distinct modes of cell division. Finally, we consider the possibility that early calpain functions may have been linked to the formation and function of MT arrays in flagella and cilia, from which later roles in cytokinesis might have evolved. This model is intended as a testable framework for future studies of calpain function across eukaryotes.

## 1. Introduction

The growth and development of all multicellular organisms depend on cell divisions that are controlled by the cell cycle. Progress through the cell cycle is directed by cyclin-dependent-kinases (CDKs) that initiate DNA synthesis and drive the cell through the cycle. The mitotic cell cycle involves chromosome condensation and nuclear envelope breakdown, followed by mitogen-activated protein kinase (MAPK)-mediated attachment of the chromosomes to mitotic spindle fibers, and finally sister chromatid separation and physical division of the cytoplasm and organelles by cytokinesis. A key stage of mitosis is the organization of microtubules (MT) into the mitotic spindle by the animal centrosome or plant microtubule organizing center (MTOC), cytoskeletal components that orient the chromosomes along a set division plane to establish the subsequent division and new cell wall position (Table 1).

**Figure 1:**
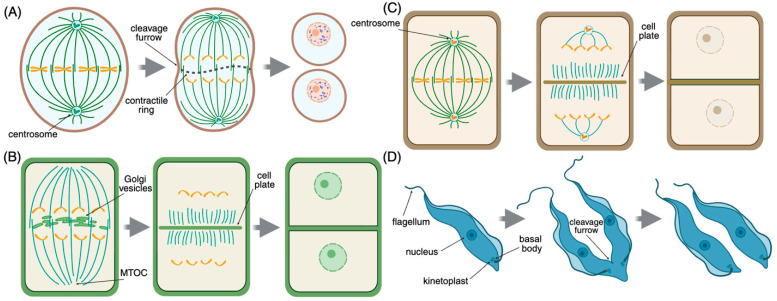
Schematics illustrating the process of cytokinesis in the somatic cells of various organisms. (**A**) Cytokinesis in animal cells. (**B**) Cytokinesis in land plant cells. (**C**) Cytokinesis in brown algal cells. (**D**) Cytokinesis in trypanosome cells. Figure created in BioRender. Fletcher, J. (2026) https://BioRender.com/5eqwv1p.

Several cell division mechanisms occur in eukaryotes. In animals, the cell division plane is determined by the position of the mitotic spindle anchored at the opposite ends of the cell by the centrosomes, consisting of two centrioles that organize the MT as the main MTOC. Cytokinesis then occurs through the formation of an actomyosin contractile ring that constricts the cell membrane to form a cleavage furrow that divides the cell in two across the short axis (Figure 1A). This ancient evolutionary process developed before the split of the eukaryotic supergroups [27,28].

In most land plant cells, at the onset of mitosis, the location of the cell division plane is set by the preprophase band (PPB). The PPB is a transient cortical ring of MT, actin filaments, endoplasmic reticulum, and associated proteins that is assembled and oriented perpendicular to acentrosomal MTOC activity and encircles the nucleus [29,30]. The PPB leaves a positional cue at the future cell division site that guides the activity of phragmoplasts, assemblies of MT and Golgi vesicles that are organized by MTOC activity and fuse to form a cell plate. The cell plate then expands outward into a new cell wall that separates the daughter cells (Figure 1B). This group of organisms lacks centrosomes, and cell division depends on guidance by distributed MTOCs. Interestingly, the somatic cells of the basal liverwort plant *Marchantia polymorpha* initially form centrosome-like structures called polar organizers that appear before the PPB and serve as the MTOC [18]. The cells subsequently divide like those of higher plants under direct guidance of the PPB by forming a cell plate [31]. This dual mode of cytokinesis utilizing both centrosome-like structures and PPB is suggested to exemplify a stepwise evolutionary transition from green algae to land plants [19] and is important to enable the asymmetric cell divisions that are especially important to meristem function, branching, and setting 3D tissue orientation [32]. Like land plants, most fungi also lack centrosomes, and their cell divisions are controlled by a MTOC named the spindle pole body (SPB) that plays a role in MT nucleation and additionally contributes to nuclear envelope breakdown [33].

Brown macroalgae, which are part of the SAR supergroup consisting of Stramenopiles, Alveolates and Rhizarians, have a combined set of structures, and their cell division occurs via a combination of the two processes. That is, like animals, brown algae contain centrosomes that act as the MTOC [34]. Yet, rather than forming a contractile ring and furrow, they divide by centrifugal growth and Golgi vesicle-mediated fusion of wall material from inside to outside to form a cell plate [35,36], similar to land plants [37] (Figure 1C). This division pattern is likely explained by the fact that, like plants, brown algae harbor physiologically important cell walls allowing especially large kelps such as *Nereocystis luetkeana* and *Macrocystis pyrifera* to grow impressive forest canopies, which depends on new cell wall deposition to complete cell division and grow large structures [38]. However, brown algae lack the cortical MTs, PPB and phragmoplasts present in land plants [39,40].

A fourth eukaryotic supergroup is the Excavata group of unicellular, mostly flagellated protists. Although it is debated whether this group is truly monophyletic [41], Excavates are generally characterized by their asymmetrical, “excavated” feeding grove and many undergo a specialized cell division by longitudinal fission in which the parent cell splits lengthwise in two. This process is well described in the human parasite *Trypanosoma brucei*, in which basal bodies that anchor the flagella of the cell serve as centriole-like structures [42]. The basal body is a MT-composed organelle that, like a centriole, has a core ninefold-symmetrical MT and cartwheel structure and is physically connected to a kinetoplast consisting of mitochondrial DNA (Figure 1D). At the onset of mitosis, the basal body replicates, and the new basal body assembles a new flagellum, the tip of which defines the position where cytokinesis initiates [43]. Cytokinesis then proceeds via ingression of a cleavage furrow along the longitudinal axis of the cell from anterior to posterior [42,44,45], without the formation of a contractile ring at the cleavage furrow site [46]. In all these cases, the positioning of the MTOC is critical for cytokinesis to proceed in the proper plane and for the correct partitioning of nuclei and organelles into the two daughter cells.

Calcium affects nearly every aspect of life by linking external stimuli to intracellular events via movement through ion channels or by release from cellular stores [47,48,49]. Changes in calcium concentrations are universal regulators from bacteria to animals, evolutionarily identified across early eukaryote linages before the split leading to different phylogenetic groups of plants, brown algae, fungi, and animals [50]. Calcium plays a crucial role throughout the cell cycle in nuclear envelope breakdown, chromosome segregation, and cytokinesis [51]. In fission yeast and animals, calcium spikes at the onset of cytokinesis initiate cleavage furrow ingression and subsequent plate formation [52,53,54]. At the molecular level, calcium is linked to the cell cycle by motility and motor proteins such as dynein and kinesin, RhoGTPases, initiation of the MAPK cascade, and the cytoskeleton [50,55,56]. Despite its pivotal role, a possible universal link remains to be detected between calcium and the many biological functions it regulates in most living organisms.

The transport of calcium into cells occurs via mechanosensitive channels: force-sensing integral membrane proteins found in all kingdoms of life that are activated by mechanical stimuli exerted on cell membranes [57]. Among these channels, Piezo1 plays an important role in regulating the cell cycle from centrosome integrity through divisions until cell death [58]. In mammals, activated Piezo1 has been shown to anchor cytoskeletal components and function upstream of calpains [59,60,61,62], calcium-activated cysteine proteases that are found in some bacteria and nearly all eukaryotes [20]. Calpains are central to many fundamental cellular processes, including cytoskeletal remodeling, cell signaling and apoptosis, centrosome functions, centrosome duplication and positioning, MT polarization, connecting chromosomes to spindles, chromosome positioning, and cell cycle progression [63,64,65,66]. Mammalian calpains are grouped into classical and non-classical calpains based on their domain organization [5]. The classical calpains contain a core CysPc (PC1 and PC2) domain as well as C2, calpain-type β-sandwich (CBSW), and penta-EF hand (PEF) domains [2], whereas the non-classical calpains contain additional zinc finger (Zn), microtubule interacting and transport (MIT), and WW domain combinations [6,67]. All vertebrate and insect calpains are cytosolic proteins that function in the cytoplasm and are not anchored to cell membranes, although they are associated with membranes and in some cases this association is linked to their activation [6,68]. In mammals 14–15 genes encode calpain proteins, some of which are specific to certain tissues, whereas others are ubiquitous [69,70].

In contrast, plants, including primitive mosses and liverworts, contain a single unique calpain called DEFECTIVE KERNEL1 (DEK1). Unlike animal calpains, at its amino terminus the DEK1 protein contains an extensive membrane anchor consisting of 21–24 predicted transmembrane domains interrupted by an unstructured loop or channel domain [69,71,72]. This is followed by an intracellular linker containing a LamininG-like domain (LGL) and the calpain protease comprised of a CysPc and a CBSW domain [71,73]. The *DEK1* gene is highly expressed in actively dividing cells, including stem cells [74,75,76], and the respective model *Zea mays* and *Arabidopsis thaliana* proteins have been found to be localized at the plasma membrane, endoplasmic reticulum (ER), and in endosome-like compartments [69,77,78]. During 3D growth in the moss *Physcomitrella patens*, the PpDEK1 protein exhibits more polarized localization restricted to the plasma membrane between recently divided cells [72]. Multiple studies have shown that DEK1 is essential for plant embryogenesis and post-embryonic development, epidermal identity, cell-to-cell adhesion, 3D cell orientation, stem cell maintenance and gene regulation, microtubule orientation, and cell wall positioning [71,74,75,76,77,78,79,80]. These diverse biological roles have led previous investigators to conclude that DEK1 has a variety of activities within the cell [79]. Here, we consider whether these activities as well as those of animal cytosolic calpains could be linked by a single core function involving calcium-responsive regulation of membrane-cytoskeleton interactions during cell division.

## 2. Methods

### 2.1. Brown Algae Genome Mining for Calpain Sequences

To identify putative DEK1-like calpains in brown algae, the Phaeoexplorer brown algae protein database [81] was queried using the default parameters of BLASTp with a DEK1-like calpain sequence from *Ectocarpus* sp. 7 (Ec-01_007845.1). We selected the top 100 hits from the BLASTp search that aligned to the *Ectocarpus* CysPC domain sequence and contained at least one amino acid of the catalytic triad, thereby capturing both sequences with high overall amino acid similarity to our query as well as partial sequences with lower amino acid similarity from less well-annotated brown algal genomes. The domain compositions of these top 100 hits were predicted individually using InterProScan 5 [82], and the output filtered to exclude sequences lacking a predicted CysPC domain. To visualize and compare the domain compositions of the putative brown algal calpains, this filtered output file was uploaded to RStudio Version 2025.05.1+513. Additionally, the 10 available brown algal proteomes and 26 red algal proteomes in the PhycoCosm database [83] were individually queried using BLASTp with the same DEK1-like sequence from *Ectocarpus* sp. 7. The domain compositions of the top hits from these queries were predicted using InterProScan. The output was filtered to exclude sequences lacking a predicted CysPC domain, and the remaining sequences uploaded to RStudio. To generate the brown algal calpain domain composition schematic in RStudio, InterProScan domain prediction outputs were first manually reformatted in Excel for compatibility with the drawProteins package [84]. Extraneous annotations and structural descriptions were filtered out, and entry names were optimized for clarity. The ggplot2 package was then used to construct the initial plot, followed by domain architecture visualization using drawProteins. The BiocStyle and knitr packages in RStudio were employed to visually optimize and format the figure.

### 2.2. Multiple Sequence Alignment and Phylogenetic Analysis

To probe the composition of the brown algal CysPC domain, a sequence alignment was generated using canonical CysPC domains from plants and cyanobacteria alongside selected DEK1-like sequences from heterokonts, including brown algae, as identified by Denoeud and coworkers [81]. Cyanobacterial sequences were obtained from Veselenyiova and coworkers [2], and the *Physcomitrella patens* sequence was downloaded from the reference genome in NCBI GenBank [73,85]. *A. thaliana* and *Zea mays* sequences were downloaded from Uniprot [86,87]. Sequences were aligned by Multiple Alignment using Fast Fourier Transform (MAFFT), followed by automatic trimming using TrimAI [88,89]. Following the generation of a trimmed alignment file, the sequences were visualized using JalView 2.11.5 [90]. To generate the phylogenetic tree, the trimmed alignment file was filtered to include only the heterokont sequences from the Phaeoexplorer database. This alignment file was then loaded into PhyloSuite v2.0dev2 and a treefile generated with the IQ-TREE plugin using the Auto model, with *Schizocladia ischiensis* included as the outgroup [91,92,93]. Bootstrap values were calculated with 1000 replicates. The resulting treefile was uploaded to the ITOL webserver [94] where the final phylogenetic tree was visualized and formatted. Entry names were optimized for clarity.

## 3. Results

### A Unifying Hypothesis for Calpain Involvement in Cytokinesis

Here we present our unifying hypothesis that calcium-regulated calpains may contribute to mitosis and cytokinesis by modulating microtubule (MT) organization, cell division plane positioning, and cytokinetic progression. This model-generating hypothesis is based on correlations among calpain domain architecture, localization, calcium signaling, cytoskeletal organization, and cytokinesis phenotype datasets from the literature (Table 1). It is intended as a framework for community testing rather than as a demonstrated general mechanism and should not be interpreted as evidence that calpains directly anchor MTOCs or centrosomes in all organisms. Rather, we propose calpain function in MTOC/centrosome positioning as a possible unifying mechanism that can be experimentally tested against alternative explanations, such as multiple indirect roles for calpains in cytoskeletal remodeling, membrane trafficking, mechanotransduction, cell wall deposition, and proteolytic regulation of mitotic factors.

**Figure 2:**
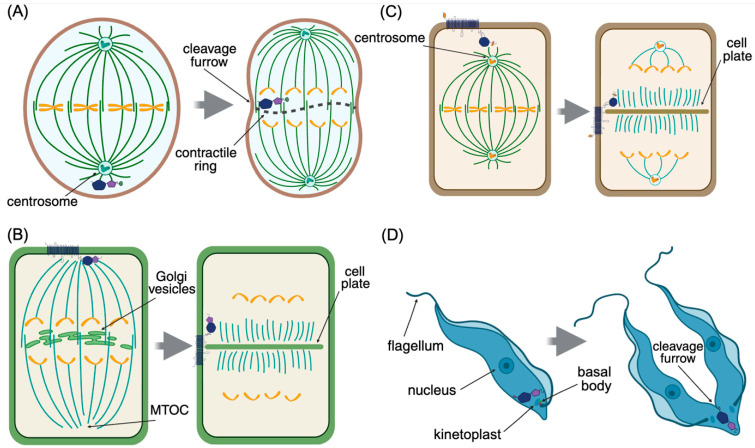
Schematics illustrating the proposed, hypothetical activities of calpains during mitosis and cytokinesis in different types of organisms. (**A**) Possible cytosolic calpain functions during cytokinesis in animal cells. (**B**,**C**) Possible transmembrane calpain functions during cytokinesis in (**B**) land plant cells and (**C**) brown algal cells. (**D**) Possible cytosolic calpain function during cytokinesis in trypanosome cells. These diagrams represent a model-generating hypothesis and should not be interpreted as evidence that calpains directly anchor centrosomes or MTOCs in all systems. Figure generated using Created in BioRender. Fletcher, J. (2026) https://BioRender.com/5t2jira.

In animals, cell division is mediated by the main centrosomal MTOC positioning that directs MT spindle formation and then sets the position of the cytokinetic contractile ring, which then forms the furrow that divides the cell. We propose that cytosolic calpains set the centrosome position, thereby directing and facilitating the progress of furrow ingression and enabling the completion of cell division at the proper position (Figure 2A). This activity may occur in response to calcium pulses that coincide with the initiation of furrow ingression [52,54] and may involve CysPc-mediated degradation of proteins such as RhoA and fodrin [95,96], which are confirmed targets of calpain-mediated cleavage [49,97,98]. RhoA is a small GTPase that, when activated, stimulates actin nucleation and myosin activation to form the contractile ring and is sufficient for furrow initiation [96,99]. Fodrin is a non-erythroid form of spectrin that functions in metazoans to nucleate MT from centrosomes, elongate spindles, and position chromosomes at the metaphase plane, and is required for mitosis progression along with kinesins and dyneins [70,100,101,102]. Thus, the available data are consistent with, although not evidence of, the hypothesis that cytosolic calpains participate in MTOC/centrosome-linked cell division plane control.

Alternatively, such activity could occur via multiple distinct calpain activities, such as proteolytic regulation of cytoskeletal or membrane-associated substrates, interactions with microtubule-associated proteins, or indirect signaling pathways activated by cytokinetic calcium pulses [52,54]. In this scenario, calpains would not necessarily serve as physical anchors for centrosomes. Instead, they might help regulate the local protein complexes that connect calcium signaling, cytoskeletal remodeling, and membrane deformation during cytokinesis.

In land plants, cytokinesis is initiated by two MTOC anchored at the opposite ends of the cell and their interconnected MTs which perpendicularly orients the PPB. The PPB positions the final cytokinetic division by the phragmoplast that directs cell plate formation and deposits cell wall material between the two newly forming daughter cells. The membrane-anchored calpain DEK1 is not required for PPB formation but is necessary for the arrangement of MTs into organized patterns and to correctly position the PPB [76]. Therefore, we hypothesize that DEK1 may act to anchor the MTOCs and organize the subsequent positioning of the CMTs and PPB (Figure 2B), setting the new cell wall position and also facilitating the deposition of new cell wall material at the correct position by the phragmoplasts [103]. We propose this could take place through direct DEK1 interactions with MT-associated proteins, mechanosensitive calcium signaling, or regulation of proteolytic substrates. Yet, we recognize that at present there is no direct evidence that DEK1 physically binds or anchors plant MTOC activity, and therefore DEK1-mediated MTOC or MT anchoring is only one possible core mechanism that could explain the observed developmental and cytoskeletal phenotypes. An alternative explanation is that it could occur indirectly through independent effects of DEK1 on membrane trafficking, cellulose synthase behavior, or cell wall mechanics.

**Figure 3:**
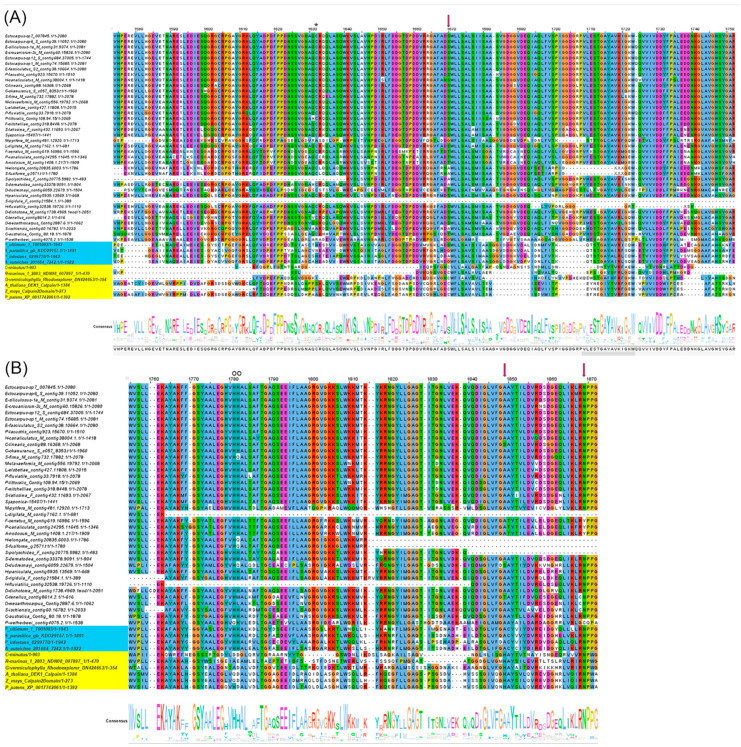
Multiple sequence alignment of the core CysPC domain of calpain sequences from brown and red algae as well as selected land plants and mosses. Alignment of (**A**) The PC1 subdomain and (**B**) the PC2 subdomain. Conserved amino acids are highlighted in colored boxes. Red arrows indicate the position of the amino acids in the catalytic triad (Cys-His-Asn). The asterisk (*) indicates the position of the highly conserved cysteine residue in the brown algal sequences. The two open circles positioned above the 1780 at the top of panel B Open circles indicate the positions of the highly conserved histidine residues in the brown algal and oomycete sequences. Oomycete species are highlighted in blue, and red algal, land plant and moss species are highlighted in yellow. The consensus amino acid sequence is shown along the bottom.

Brown algal somatic cell division includes a combination of features that occur separately in animals and land plants. Brown algal mitosis depends on centrosomes that act as MTOCs, like animal cells, followed by plate formation to set the new cell wall, like land plant cells (Table 1). This combination makes brown algae a good model system for assessing whether membrane-associated calpains might be involved in coordinating centrosome-associated MTs with cell plate formation. Until recently, it was not feasible to probe the calpain distribution among the diverse families of brown algae, but the release of several dozen high-quality brown algal genomes [81,104,105] has enabled this type of investigation. We used the conserved CysPc domain amino acid sequence from *A. thaliana* to identify calpain sequences across brown algal genomes. We identified a single amino acid sequence in each brown algal genome that aligns well with conserved CysPc domain amino acid sequences from oomycetes, land plants, moss, and fungi (Figure 3). This finding suggests that DEK1-like calpain sequences are broadly represented among brown algal genomes. Yet, because genome complexity, gene annotation, and search sensitivity vary among species, our results do not prove that all brown algae contain only a single calpain gene. Future HMM-based searches and reciprocal sequence comparisons using improved genome annotations will be needed to rule out the presence of more divergent or fragmented calpain-like sequences.

The CysPc domain consists of the PC1 (Figure 3A) and PC2 (Figure 3B) domains and contains three amino acids—cysteine, histidine, and asparagine—that make up the catalytic triad in canonical animal and land plant calpains [106,107]. Among the brown algal CysPc sequences, the asparagine residue in the PC2 domain is conserved in the majority of the brown algae species examined, but has been substituted with a tyrosine residue in *Fucus serratus*, *Pelvetia canaliculata*, and *Ascophyllum nodosum*, three members of the Fucaceae family (Figure 3B). The histidine residue in the middle at position 1848 in the PC2 domain is not conserved in any brown algal or oomycete species in our dataset, having been replaced with an alanine, serine, or threonine residue (Figure 3B). Each brown algal and oomycete sequence instead features one or two adjacent histidine residues at positions 1781-82 in the PC2 domain (Figure 3B). The cysteine residue at position 1670 in the CysPc domain also is not present in any brown algae calpain sequence identified, having been substituted with a serine residue in many of them and by a threonine, alanine, or glycine reside in the others (Figure 3A). This cysteine residue is likewise replaced by a serine, alanine, or glycine residue in the PC1 domain of oomycete calpain sequences (Figure 3A). However, nearly all of the brown algal calpain sequences contain a cysteine residue at position 1632, and the oomycete calpain sequences contain a cysteine residue at position 1630 (Figure 3A). Taken together, these data indicate the presence of conserved calpain-like CysPc domains in brown algae. It remains to be determined whether these proteins retain protease activity via their non-canonical catalytic triad arrangements or function partly or fully through non-proteolytic mechanisms.

**Figure 4:**
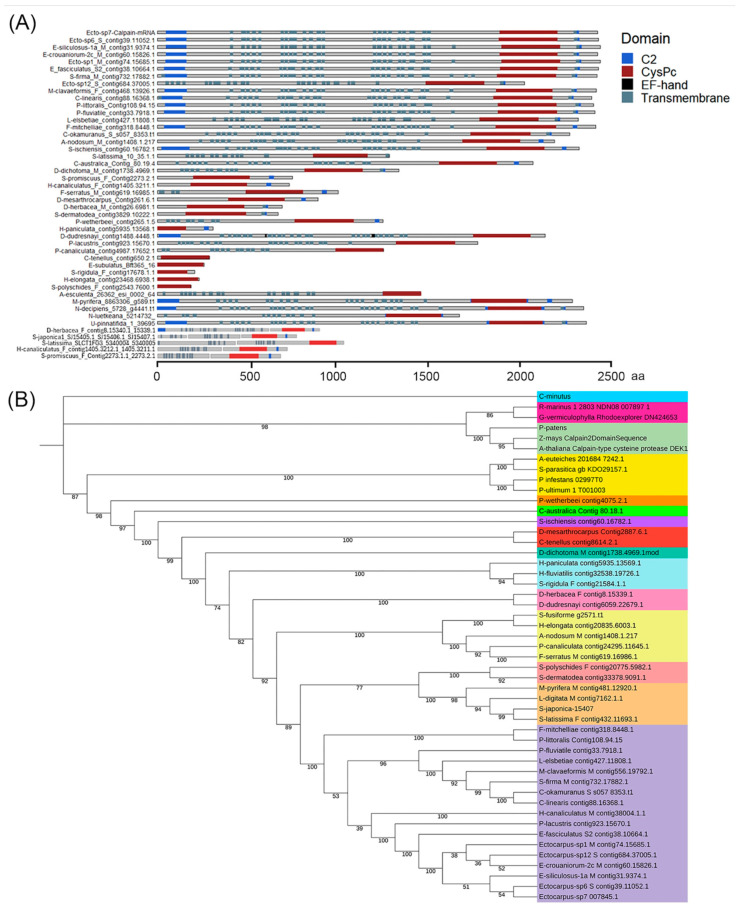
Calpain sequences in brown algae. (**A**) Domain composition of brown algae calpain amino acid sequences. The calpain sequences in five of the brown algae are discontinuous and are shown with the corresponding sequence gaps. (**B**) Phylogenetic tree of the brown algae calpain sequences. The tree was constructed using the IQtree program embedded in PhyloSuite version v2.0dev2 was used. Bootstrap values on each node indicate the proportion of recovered nodes out of 1000 bootstrap replicates. Bright blue indicates *Chroococcus minutus*, purple indicates red algal species, pale green indicates land plant and moss species, yellow indicates oomycete sequences, and dark blue indicates *Schizocladia ischiensis*. The other colors indicate species within the same brown algal order.

Most of the identified brown algal calpain-like sequences contain at least one predicted calpain domain in addition to the CysPc core. However, in five brown algae genomes only a CysPc domain is predicted (Figure 4A), likely due to incomplete sequence information or annotation. The calpain sequences of two additional species, *Discosporangium mesarthrocarpum* and *Halopteris paniculata*, are annotated as having a CysPc domain and a short C2 domain near the carboxyl terminus and may also be incomplete. We predicted the calpain sequences of *Saccharina latissima*, *Saccharina japonica*, *Scytosiphon promiscuus* and two other species based on several adjacent contigs (Figure 4A). The calpain sequences in these five and the majority of the other brown algal species are predicted to contain a series of transmembrane (TM) domains amino-terminal to the CysPc domain, and 18 species across several families are predicted to contain an additional C2 domain close to the amino terminus of the predicted protein (Figure 4A). Unexpectedly, the calpain sequence from *Desmarestia dudesnayi* uniquely harbors two EF hand domains nested among the TM motifs, which are present in some metazoan calpains but are absent from land plants [70]. Phylogenetic analysis of the brown algal calpain full-length amino acid sequences suggests the presence of a single calpain sequence in each brown algae genome (Figure 4B), as well as strong sequence conservation within the various brown algal families represented. The domain combinations identified further suggest that many brown algal calpain-like proteins are membrane-associated and potentially calcium-responsive. Although domain architecture alone cannot establish cellular function, based on their structural similarity to DEK1, we propose that these transmembrane calpains may likewise contribute to centrosome-associated MT organization and/or cell plate positioning in brown algae—a hypothesis that remains to be tested experimentally.

Beyond the calpain-like sequences identified in brown algal genomes, our searches of red algal genome and transcriptome resources using the conserved CysPc domain amino acid sequence from *A. thaliana* returned hits from *Rhodosorus marinus* and *Gracilaria vermiculophylla*. The CysPc domain of both respective red algal calpain sequences contains the Cys-His-Asn catalytic triad in the same positions as in the canonical green algal and land plant CysPc domains (Figure 3). Overall, the predicted *Rhodosorus marinus* calpain protein features a GRF zinc finger Zf_GRF-CycPc domain configuration also present in green algae, phytoplankton, and all *Plasmodium* species within the Alveolates [1,11]. The predicted *Gracilaria vermiculophylla* calpain protein features a CysPc-CBSW domain architecture present in nearly all eukaryotic supergroups and considered to be one of the ancient calpain types [1]. These findings indicate that calpains are present across the red and green algal and land plant lineages of the Archaeplastida supergroup. Broader genome sampling and experimental validation will be required to determine how widely they are distributed and whether they have cytokinetic, cytoskeletal, or other cellular functions.

Our hypothesis that calpains play a primary role in cell mitosis by positioning the MTOC and the cleavage furrow or PPB, and thereby orienting the cell division plane, is compatible with known functions for these proteins in the animal cell division process. Cytosolic calpains have already been shown to function in MT-associated processes during mammalian mitosis. In human HeLa cervical cancer cells, calpain-2 catalytic subunit (CAPN2) protein levels are elevated during mitosis, and this activation is necessary for proper MT attachment to kinetochores and correct alignment of the chromosomes at the spindle pole prior to their segregation into the two daughter cells [63]. In human breast cancer cell lines, CAPN2 plays a key role during the progression of mitosis and cytokinesis by reducing the levels of the LIM Kinase-1 (LIMK1) protein that phosphorylates the actin-severing protein cofilin-1 (CFL1) at critical steps of mitosis [108]. A similar mechanistic role for calpains in indirectly modulating the activity of MT binding proteins during mitosis can likewise be envisioned. CAPN2 is also known to degrade fodrin [97,109], which in brain cells co-localizes with MTs and plays a role in MT spindle organization and mitotic progression by associating with γ-tubulin to promote its transport to centrosomes for MT nucleation [100,101]. Fodrin concentrations peak prior to the initiation of mitosis, while the proteins dissociate from the centrosomes after prophase [100]. It has been hypothesized that calpains mediate the cleavage of fodrin from γ-tubulin to ensure the proper progression of mitosis [100]. Thus, fodrin may be a key target of cytosolic calpain activity during mitotic microtubule nucleation in metazoans, where it is predominantly found [110].

The *CAPN3* gene is expressed in skeletal muscle and encodes a canonical calpain, calpain-3. Defects in this gene cause several types of limb-girdle muscular dystrophy (LGMD) in humans. Calpain-3 has also been shown to be a functional constituent of the centrosome, and absence of the protein results in chromosome amplification and mis-orientation as well as impaired MT nucleation in myoblast cells [66]. Based on these phenotypes, Winter and colleagues propose that calpain-3 may play an additional role in cancer by “generating a permissive environment for tumor establishment.”

In addition, some non-canonical mammalian calpains contain a microtubule interacting and trafficking (MIT) domain that is absent from calpains found thus far in bacteria and tetrahymena [1,70]. The MIT domain forms an asymmetric three-helix bundle structure that resembles the first three helices in a tetratricopeptide repeat (TPR) motif [13,111]. The MIT domain can bind to ESCRT-III proteins that constrict membranes and mediate fission, including during cytokinesis where they mediate abscission of the dense central array of MTs called the midbody to create the two daughter cells [112]. During cytokinesis in humans, the ESCRT-III subunit IST1 recruits CAPN7 proteins to midbodies, where its proteolytic activity is required for abscission checkpoint maintenance and at the final stage of cell division to complete the abscission process [113,114].

*CAPN6* encodes another non-canonical mammalian calpain, calpain-6, one that lacks an MIT domain as well as the active-site cysteine residues necessary for protease activity [115]. It functions as a microtubule-stabilizing protein that associates with microtubules via its CBSW domain and colocalizes to microtubule structures, including the central spindle and midbody, during cytokinesis [116]. During mitosis, calpain-6 is associated with the mitotic spindle and appears to be required for the progression and completion of cytokinesis, similar to fodrin, as its over-expression slows the ingression of the cleavage furrow in human cells [101,116]. Taken together, these findings are consistent with our hypothesis that calpains may have a core function in MTOC/centrosome positioning during cell division, although the data do not uniquely support MTOC/centrosome anchoring as the causal mechanism.

In contrast to the body of understanding of cytosolic calpain activity during the animal cell cycle, the cellular mechanism of DEK1 calpain function remains poorly understood. DEK1 is localized to the plasma membrane as well as internal membranes [78] and regulates cell wall composition and structure [117,118,119,120], including the synthesis of cellulose and pectin and the mechanical properties of the primary cell walls [121]. Further, DEK1 is suggested to facilitate mechanosensitive calcium transport through the association of its TM domains with a yet unidentified rapid mechanically activated (RMA) channel in the plasma membrane [122]. This RMA channel displays electrophysiological properties similar to those of mouse Piezo channels [123] and may be encoded by a possible unidentified plant *PIEZO* gene or a different protein with a similar function. Piezo has 24 TM domains, comparable to DEK1’s 23, and the two proteins have seemingly shared functions [124]. Piezo1 and 2 localize to the centrosome and play roles in cell cycle progression [58] and additionally are localized to the cleavage furrow, controlling final cell division [125].

One plausible mechanism for DEK1 activity in cytokinesis is that perception of mechanical stresses in the plasma membrane from the internal tension of the dividing cell leads to transiently elevated calcium transport either through the DEK1 loop/channel structure, which itself shows properties of mechanosensory channels [103,124], or via a separate RMA channel such as Piezo1. This might then cause an increase in cytosolic calcium concentration that triggers DEK1 auto-activation [78] to facilitate the cytoskeleton positioning the MTOC and orient the new cell wall. Considering that the protease-deficient human calpain-6 co-localizes to microtubule bundles via its CBSW domain and promotes their formation and stabilization [116], we suggest that DEK1 could act through its CBSW domain to directly bind and stabilize the MTOC to set the new cell division plane.

Although alternative mechanisms for DEK1 function currently cannot be excluded, our hypothesis is consistent with multiple lines of evidence regarding DEK1 activity within cells. First, DEK1 signaling appears to be cell autonomous even though the protein is associated with the plasma membrane, ER, and endosome-like structures [77,78,126]. Second, DEK1 affects cell wall orientation, where PPB and cell walls are incorrectly positioned in severely affected *A. thaliana dek1* early embryos [76]. Third, cortical MTs in *dek1* protoderm-like cells are oriented more randomly than in wild-type cells [76]. An altered cortical MT arrangement was also documented in the epidermal cells of partial loss-of-function *dek1-4* plants [118]. These data indicate that DEK1 is important for the proper arrangement of the cortical microtubule systems, which in turn may affect the orientation of the phragmoplasts during mitosis and subsequent cell wall deposition. Several studies in *A. thaliana* and moss consistently indicate a role for DEK1 in position-dependent cell wall orientation [76,117,127]. In addition, the expression of multiple genes related to the MTOC/centrosome is altered in *A. thaliana dek1* mutants or over-expression lines, such as Human *CENP-E* and animal *KID*, *MAP65*, mitotic kinesin, *NEK5*, *CDKs*, *RanGAP*, *E2F* and *ROP* genes [76]. Many of these genes play prominent roles in regulating phragmoplast MT dynamics during cytokinesis [128]. Similarly, moss DEK1 target genes include those involved in reorientation of the phragmoplast and the cell division plane [79].

Direct targets of DEK1 protease activity have yet to be identified, but several candidate pathways could connect DEK1 to cell division and cell wall placement. Potential substrates or downstream factors include NAC WITH TRANSMEMBRANE MOTIF 1 (NTM1), a membrane-bound NAC domain transcription factor that is activated by proteolytic cleavage to mediate signaling by the plant hormone cytokinin during cell division in *A. thaliana* [129]. DEK1 also has been proposed to affect cellulose synthase trafficking and mobility at the plasma membrane, either directly by regulating CELLULOSE SYNTHASE (CESA) complexes at the post-translational level or indirectly through interactions with CESA regulatory proteins or cytoskeletal components that guide CESA movement [6]. Candidate factors include the endo-1,4-beta-glucanase KORRIGAN (KOR) that is involved in CESA regulation [130], CSI1/POM2 that facilitates binding between CESA complexes and cortical MTs [131,132], and PATROL1 (PTL1) that interacts with CSI1/POM2 and exocyst complex proteins to deliver CESA complexes to the plasma membrane [133]. These candidate pathways illustrate how DEK1 could influence cell division plane orientation through direct or indirect effects on MTs, membrane trafficking, and cell wall formation.

Together, the current evidence indicates that DEK1 is important for proper cortical MT organization and cell wall positioning and supports a connection between DEK1 and cytokinesis-related regulatory networks. It does not demonstrate that DEK1 directly binds or positions the plant MTOC, nor does it identify the underlying mechanism. It is consistent with, although not proof of, a direct role in MTOC anchoring and/or MT stabilization. Alternative explanations, including effects on cell wall mechanics, cellulose synthase movement, membrane trafficking, and/or transcriptional regulation, remain plausible. Further investigation to identify DEK1 substrates, interaction partners, and cell cycle-dependent localization patterns will be essential for resolving these alternatives.

## 4. Discussion

Calpains are calcium-dependent cysteine proteases crucial to eukaryotic cellular processes including aspects of cell cycle activity, cytoskeletal remodeling, membrane dynamics, and developmental regulation. A vast array of calpain architectural combinations exist in eukaryotes, likely originating from the domain shuffling of four combined variants early during eukaryotic evolution [1]. These domains were present in eubacteria and archaea before being combined with the core CysPc domain that defines a calpain based on its catalytic activity. Extant unicellular protists and streptophytes contain long membrane-anchored calpains, and the ciliate *Tetrahymena* genome encodes up to an impressive 26 calpain genes [5], suggesting that calpains have long been associated with cellular organization in diverse eukaryotic contexts.

Here we put forward the hypothesis that calpains may have a common role in linking calcium signaling to microtubule-associated cytokinetic processes. This hypothesis predicts that the core cellular activity of calpains is to regulate cytokinesis by coordinating MTOC and/or centrosome-associated microtubules with the spatial positioning of cytokinesis and the new cell wall. This hypothesis is speculative but more parsimonious than the broader interpretation that calpains perform multiple independent cellular functions in cytoskeletal remodeling, cell signaling, cell cycle progression, centrosome-related functions, MT polarization and apoptosis. Our proposition therefore should be read as a model-generating hypothesis, not as a theory of a universal calpain mechanism. A key goal is to define experiments that can distinguish a single calpain-mediated direct MTOC/centrosome positioning function from alternative functions in multiple processes.

Mitosis in brown algae occurs through a combination of the processes observed in plants and animals: organization of centrosomes into MTOCs, followed by cell plate and wall formation. This makes brown algae particularly relevant for assessing whether transmembrane calpains could participate in coordinating centrosome-associated MTs with cytokinetic membrane and wall deposition. Previous work revealed the presence of a single DEK1-like TM calpain sequence in the reference genomes of 17 brown algae species spanning multiple families [81]. Our analysis expands this dataset across additional brown algal species and identifies conserved calpain-like CysPc and other domains in the predicted proteins. We find that the genomes of the more than three dozen brown algal species investigated each contain a CysPc domain sequence consisting of both the PC1 and PC2 regions (Figure 3), although the domains in some species are incomplete likely due to lower genome sequence quality. Our analysis likely underestimates the true number of brown algal genomes that contain a calpain sequence, because we manually removed sequences that lacked a full length CysPC domain even if they displayed significant amino acid alignment. Further complete sequencing and annotation of brown algal genomes will provide a more comprehensive picture of the nature of the calpain family in this important group of marine Stramenopiles.

Interestingly, the positioning of the Cys-His-Asn conserved active site residues comprising the catalytic triad is not conserved in the brown algal calpain sequences. Instead, all but three of the brown algal calpain sequences contain an asparagine residue at the conserved position at the end of PC2, but have a cysteine residue at a more amino-terminal position in PC1 (Figure 3A) and one or two histidine residues at a more amino-terminal position in PC2 (Figure 3B). A parsimonious interpretation of our data is that these calpains may lack protease activity, similarly to the mammalian calpain-6 [134,135]. However, some animal calpains with substitutions in these residues do not display loss-of-function phenotypes [136], suggesting either that protease activity is not completely abrogated or that these calpains may have functions beyond proteolysis, such as in signal transduction and/or gene regulation. On the other hand, we note that the positions of the cysteine and histidine residues in the CysPc domains of brown algae calpains are nearly identical to those in oomycetes (Figure 3). The conservation of spacing of these key amino acids within these Stramenopile lineages that shared a last common ancestor over 400 million years ago may indicate that the brown algal and oomycete calpains contain a functional but non-canonical catalytic triad. Further biochemical experiments will be required to determine if this non-canonical triad retains protease activity in vivo.

Analysis of the domain architecture of brown algal calpain sequences revealed that each contains a CysPc domain consisting of PC1 and PC2 along with one or more C2 domains and several stretches of multiple TM domains, as many as 26 in total. The putative calpain sequences identified in a few brown algal species currently lack TM or C2 motifs. However, because only a single CysPc core calpain domain sequence is present in any brown algal genome (Figure 3 and Figure 4), the most parsimonious interpretation of these data is that the sequences are incomplete and that the full domain configuration is likely to be C2-TML-CysPc-C2. Although C2 domains are present in classical animal calpains and TM domains are present in land plant calpains, the brown algal calpain C2-TML-CysPc-C2 domain configuration is so far unique among eukaryotes [1,6]. This architecture may represent a fifth type of eukaryotic membrane-anchored calpain, most similar to the Type 3 C2-TML-Linker-CysPc-WW version found in various oomycetes [6], which like brown algae are members of the Stramenopiles subclade. The predicted *D. dudesnayi* C2-TML-EF-TML-EF-TML-CysPc domain structure is also unique, among not only the brown algae but eukaryotes in general, although an EF-CysPc configuration is present in *Tetrahymena thermophila* [1], an Alveolate member of the SAR supergroup. Further investigation will be required to discern whether these *D. dudesnayi* calpain putative EF hand motifs are functional and what their evolutionary origin might be.

Our characterization of brown algal calpain-like sequences emphasizes the modularity of these cysteine protease-related proteins across eukaryotes. Beyond the CysPc protease core domain, the C2 domain primarily acts as a calcium-binding motif that is involved in signal transduction and membrane association [6,137,138]. The combination of C2 and TM domains hints that brown algal calpain proteins may have the capacity to associate with membranes and detect and transmit calcium signals across them, although this inference is based on domain architecture rather than functional data. Their overall sequence similarity to *A. thaliana* DEK1 raises the possibility that the brown algal calpains may bind and stabilize MTs in their centrosomes during cytokinesis, an unproven but experimentally testable hypothesis for the future. Currently, we cannot rule out that the brown algal calpains have lineage-specific functions unrelated to MT positioning.

The centriole is an ancient MT-based cylindrical organelle that not only nucleates the formation of the centrosome, the main MT organizing center in animal cells, but also can be modified to form basal bodies that template the formation of cilia and flagella. The centrosome templates the formation of the primary cilium from one of its centrioles, which is then assembled at the plasma membrane [139]. There is a direct structural relationship between MT and membrane furrowing during ciliogenesis, where cilia initiate as a basal membrane invagination, via interaction with actin-regulating proteins, Rho GTPases, and formins [140,141,142,143]. In fact, the presence of centrioles correlates across the tree of life with the presence of cilia but not of centrosomes, suggesting that an ancestral role of centrioles may have been to direct cilia formation and activity [144,145].

Calcium signaling is involved in a variety of cilia-dependent biological processes, including mechanosensation, chemosensation, cell cycle control, cell polarity, and cell migration [56], and evidence exists for a role for calcium-dependent calpains in mammalian cilia and flagella. Calpains are colocalized with cilia components in multiple organisms including in the green algae *Chlamydomonas reinhardtii* flagellar proteome, in Trypanosomes associated with cilia functions as calpains are essential for flagellar attachment/structure, in sea urchins for mediating sperm activation, and in human sperm [146]. In mouse fibroblast cells, calpain-6 acts as an inducer primary of ciliogenesis that is proposed to increase the levels of alpha-tubulin as a post-translational modification to promote MT stability and function [147]. In spermatozoa, which use flagella for motility, calpain activity is essential for physiological processes such as capacitation, acrosomal reaction, mobility, and fusion that are required for successful fertilization [148,149,150,151]. Specifically, CAPN1 regulates the remodeling of the spectrin cytoskeleton [152] as well as lipid raft rearrangement and activation of the Src kinase family [153]. Membrane contractile networks are especially fundamental to cytokinesis and cell motility, depending on actin, tubulin, and MT and their crosslinking to proteins and membranes. Organization of MTs in centrosomes is common to these functions, and it is possible that these functions may all be controlled by calpains.

One plausible scenario is that early in eukaryotic evolution, calpains contributed to calcium-regulated membrane–microtubule functions associated with cilia, flagella, or basal bodies, and that related activities were later co-opted into cytokinesis and cell division plane control in different lineages. However, this is not the only possible interpretation. Given the diversity of calpain domain architectures and activities across eukaryotes, calpains instead may have undergone multiple lineage-specific co-options into overlapping cytoskeletal, membrane, signaling, and proteolytic roles. Distinguishing between a deeply conserved ancestral role and repeated lineage-specific recruitment will require broader phylogenetic sampling, functional studies in non-model organisms, sub-cellular localization of calpains in the cells of diverse eukaryotes, and comparison of calpain interaction partners across taxa.

## 5. Concluding Testable Predictions and Future Experiments

Our hypothesis both raises new questions and opens novel avenues for the investigation of calpain function at the cellular and biochemical levels. One key question is how the cytosolic calpains might set the position of the cell division plane when they themselves are not membrane anchored. The answer may lie in their association with membrane-bound proteins that have a specific sub-cellular localization, but this remains to be determined. The sub-cellular localization of TML calpains during mitosis in different cell types also warrants additional careful investigation. In addition, the proteolytic substrates of transmembrane calpains such as DEK1 are as yet unknown, and whether such TML calpains can directly bind MTs needs to be tested. The relative contribution of calpain proteolytic activity and MT anchoring activity towards facilitating MTOC orientation during cell division also remains to be assessed.

Our hypothesis also generates a number of experimentally testable predictions. First, if calpains directly participate in positioning centrosomes, MTOCs, or cytokinetic MT arrays, then specific calpains should show cell cycle-dependent localization to centrosomes, basal bodies, PPBs, phragmoplasts, midbodies, or related MT-associated structures. Live cell imaging of fluorescently tagged calpains in animal, algal, and land plant cells would provide a direct test of this prediction.

Second, if DEK1 contributes directly to MT organization during plant cytokinesis, then the protein should physically interact with MT-associated proteins, cortical MTs, or proteins involved in PPB and phragmoplast positioning. Proximity labeling, co-immunoprecipitation, and in vitro binding assays could test this possibility. If such interactions are not detected, then an indirect mechanism related to transcriptional regulation and/or proteolysis of cell division, cytokinesis, and cell wall-related genes and proteins would be favored.

Third, a broader comparative genomics analysis using more complete sequencing, improved gene prediction, HMM-based searches, and reciprocal homology tests will be needed to define the full calpain repertoire in brown and red algae and to assess whether these proteins are conserved as single-copy genes or represent only the currently detectable members of a more diverse family. Further, if brown algal transmembrane calpain-like proteins participate in cytokinesis, they should be expressed during cell division and localize near centrosomes, division planes, or sites of cell plate formation.

Fourth, perturbing brown and red algal calpain gene function should affect centrosome–cell plate coordination or cell wall placement. Loss-of-function or domain-specific mutations should disrupt cell division plane fidelity, centrosome or MTOC positioning, MT organization, or other aspects of mitosis and/or cytokinesis as they do in animals and land plants. Rescue experiments using catalytically inactive proteins, CBSW domain mutants, C2 domain mutants, or transmembrane-domain mutants would help distinguish proteolytic activity from possible structural, scaffolding, or membrane-associated functions. Conversely, the absence of cell division-stage localization or cytokinetic phenotypes would argue against our proposed cellular role for calpains in brown algae.

Finally, the catalytic status of the non-canonical brown algal CysPc domains should be tested biochemically. Purified brown algal CysPc domains could be assayed for calcium-dependent protease activity and compared with those of canonical plant and animal calpains. These experiments would determine whether the conserved but position-shifted cysteine and histidine residues form a functional non-canonical catalytic triad or whether brown algal calpain-like proteins act primarily through non-proteolytic mechanisms.

Together these experiments should allow the proposed hypothesis to be validated, modified, or rejected based on data from specific eukaryotic lineages. We hope that this article provides a useful conceptual framework for designing experiments that clarify how calpains contribute to calcium signaling, MT organization, cytoskeletal regulation and cytokinesis across diverse eukaryotes.

## Figures and Tables

**Table 1:** Comparative overview of centrosome or microtubule-organizing center (MTOC) structure types, cytokinesis types, presence of cilia and/or flagella, and calpain domain combinations across major lineages and organisms according to the literature.

Organism	Type of Chromosome Organizer (Centrosome if +)	Cytokinesis Type	Flagella/ Cilia Movement	Additional Calpain Domains Beyond CysPc [1]
Cyanobacteria [2]	NA	Fission	NA	ND
Dinoflagellate *Symbiodinium minutum*	CL	Acrobase furrow	+	44 calpain variants [3] ND
Trichomonas	CL attractophore [4]	Ventral furrow	+	TML1 [1]
Apusomad Thecamonas	BB	Binary fission furrow	+	TML1&2, CBSW, MIT [1,5,6]
Lower fungus Yeast	SPB	Furrow	+	PalB [7]
Fungus *Aspergillus nidulans*	SPB	Septation cross walls	-	CBSW, MIT, PalB [5,6,7]
Fungus Magnaporthe	SPB	Furrow	-	C2, EF [8,9]
Parasitic Alveolata SAR *Plasmodium falciparum*	Centriolar plaque [10]	Furrow	+	CBSW [6,11,12]
Rhizaria SAR Oomycete *Phytophthora infestans*	+	Cleavage furrowing	+	TML3 & 4, CBSW, MIT, C2, Zf-GRF [1,3,5,6,8,9,13,14]
Ciliate Alveolata SAR Tetrahymena	BB feeding + MTOC	Contractile ring	Cilia	TML1, TMS, EF [1,5,15]
Stramenopila SAR Brown algae (BA)	+	Centrifugal growth cell wall inside to out	+	Predicted TML, C2 [5,8,9]
BA *Desmarestia dudresnayi*	+	ND	+	Predicted TML, C2, EF [5,8,9,15]
Red algae *Rhodosorus marinus*	Ring shaped MTOCL	Furrow	-	Predicted Zf-GRF [11]
Green algae (GA) Ostreococcus	SPB	Phycoplast furrowing	-	Zf-GRF [3,11]
GA Chlamydomonas	BB/MTOC advanced MTOC close to animal	Phycoplast furrowing	+	CBSW [6]
Unicellular biflagellate charophyte GA *Mesostigma viride*	BB	Centripetal cleavage [16]	+	TML, CBSW [6,16,17]
Basal land plants Liverworts, *Marchantia polymorpha*	PO [18]	PPB	+	2 TML variants, CBSW [6,19]
Higher land plants	MTOC	PPB+	ND	TML2, CBSW [5,6]
Animals Opistochonta	+	Furrow	Flagellum	C2, CBSW, MIT, PEF [5,6,9,13,20]
Human CAPN7 most ancestral calpain	NA	NA	NA	MIT, PalBH [21]
*Drosophila melanogaster*	+	Furrow	Flagellum	PEF, SOH [22,23,24]
*Caenorhabditis elegans*	+	Furrow	Cilia	C2, MIT, PalBH, SOH [25,26]
*Homo sapiens*	+	Furrow	Flagellum	MIT, CBSW, C2, PEF, PalBH [8,13,20,25]

These entries summarize different types of evidence and should not be interpreted as having equal evidentiary status. Organismal chromosome organizer types, cytokinesis types, and cilia/flagella presence (+) or absence (-) are based on published data where available. Calpain domain combinations are based on sequence prediction, comparative genomics, or previous reports. The table is intended to show patterns that motivated the hypothesis, not to assign demonstrated cytokinetic functions to all listed calpain proteins. Abbreviations: BA: Brown algae; BB: Basal Body; CAPN: Calpain; C2: C2 domain; CBSW: calpain-type β-sandwich; CL: Centrosome-like; EF: EF-hand; GA: Green algae; MIT: Microtubule Interacting and Trafficking; MTOC: Microtubule Organizing Center; MTOCL: MTOC-like; NA: Not applicable; ND: Not detected; PalB: calpain-like cysteine protease; PalBH: PalB homologue; PEF: Penta EF-hand; PO: Polar organizer; PPB: Pre Prophase Band; SAR: Stramenopiles, Aveolates, Rhizarians; SOH: SOL homologue; SPB: Spindle Pole Body; TML: Transmembrane Long; TMS: Transmembrane Short; Zf-GRF: Glycine-Rich Zinc Finger.
